# Relationships between changes in lateral vestibulospinal tract excitability and postural control by dynamic balance intervention in healthy individuals: A preliminary study

**DOI:** 10.3389/fnhum.2023.1109690

**Published:** 2023-02-01

**Authors:** Tomoyuki Shiozaki, Yohei Okada, Junji Nakamura, Kozo Ueta, Hiroaki Tanaka, Mako Moritani, Tadashi Kitahara

**Affiliations:** ^1^Department of Otolaryngology-Head and Neck Surgery, Nara Medical University, Kashihara, Japan; ^2^Neurorehabilitation Research Center of Kio University, Nara, Japan; ^3^Graduate School of Health Sciences, Kio University, Nara, Japan; ^4^Department of Rehabilitation, Nishiyamato Rehabilitation Hospital, Nara, Japan; ^5^Department of Rehabilitation, Shiga Hospital, Shiga, Japan; ^6^Department of Physical Medicine and Rehabilitation, Kansai Medical University, Osaka, Japan; ^7^KMU Day-Care Center Hirakata, Kansai Medical University Hospital, Osaka, Japan; ^8^Department of Faculty of Medicine, Nara Medical University, Nara, Japan

**Keywords:** galvanic vestibular stimulation (GVS), H-reflex, lateral vestibulospinal tract (LVST), postural control, dynamic balance intervention

## Abstract

**Introduction:**

We conducted dynamic balance or static intervention on healthy young adults to examine the changes in lateral vestibulospinal tract (LVST) excitability and postural control that ensued following dynamic balance intervention and to investigate the correlation between these changes.

**Methods:**

Twenty-eight healthy young adults were randomly assigned to either the dynamic balance group or the control group. They performed either a dynamic balance or static intervention for 10 trials of 30 s each and were assessed for head jerks during the intervention to confirm adaptation to the intervention. The dynamic balance intervention consisted of maintaining balance on a horizontally unstable surface, whereas the control intervention involved standing in the same foot position as the dynamic balance intervention on a stable surface while completing a maze task. LVST excitability and postural stability were assessed before and after the interventions. LVST excitability was assessed as the change rate in the soleus H-reflex amplitude with galvanic vestibular stimulation (GVSH). The velocity and area of the center of pressure (COP) were examined in the eyes closed/foam rubber condition.

**Results:**

No significant main and interaction effects (task, time) were observed for GVSH and COP variables. In the dynamic balance intervention, head jerk significantly decreased, and GVSH-change and changes in head jerk and COP area were significantly negatively correlated.

**Discussion:**

The LVST excitability change for the dynamic balance intervention varied among the participants, although increased LVST excitability may have been related to increased postural stability.

## 1. Introduction

The lateral vestibulospinal tract (LVST) is a pathway for the vestibulospinal reflex and is involved in the postural control of antigravity muscles (Grillner et al., 1970; Grillner and Hongo, 1972; Molina-Negro et al., 1980). LVST excitability increases in anti-gravity posture (MacKinnon, 2018; Tanaka et al., 2021). The LVST is a pathway that projects from the peripheral vestibule to the lumbar spinal cord *via* the vestibular nucleus. The otolith organs receive linear and gravitational acceleration information from the head, and the semicircular canals receive information on the rotational acceleration of the head. In response to information from the peripheral vestibular organs, the two pathways of the LVST and the medial vestibulospinal tract cooperate to control balance in standing and gait (Molina-Negro et al., 1980; Jang et al., 2018). Postural control is highly adaptable and can be improved through balance training, and the human central nervous system responds instantaneously to changes in the support surface or alterations in the peripheral feedback (Taube et al., 2008). Vestibular inputs are essential for balance whenever support surfaces are irregular or in motion. In patients with vestibular disorders, instability of the support surface in the closed eye condition results in a delayed response of the distal muscles of the lower extremities due to their inability to adapt to changes in the support surface (Nashner et al., 1982). This is thought to suggest that vestibular stimulation modulates the muscle output of the distal lower leg muscles through the LVST when standing on an unstable floor surface. Therefore, during a dynamic balance intervention, vestibular inputs by head movements could increase, and with increased vestibular inputs, LVST excitability may increase. Repetition of a dynamic balance intervention may increase LVST excitability after the task. However, there have been no reports examining how LVST excitability changes in response to a dynamic balance intervention.

Lateral vestibulospinal tract excitability can be assessed through the use of neurophysiological techniques involving galvanic vestibular stimulation (GVS), which activates the vestibular system and LVST *via* percutaneous means (Kennedy and Inglis, 2001; Ghanim et al., 2009; Okada et al., 2018). The facilitation ratio of the H-reflex of the soleus muscle, as conditioned by cathode GVS (GVSH), serves as a reflection of LVST excitability. We have previously validated the optimal GVS stimulus intensity for the measurement of GVSH (Okada et al., 2018) and demonstrated that the method exhibits high test-retest reliability, with a minimum detectable change (MDC) in GVSH of 11.0% (Nakamura et al., 2021). Furthermore, we have observed that GVSH increases in antigravity postures, providing evidence of LVST function (Tanaka et al., 2021). However, the effect of dynamic balance interventions on GVSH has yet to be clarified, with the changes in GVSH having only been studied in the context of gaze stabilization exercises and noisy GVS following cerebellar repetitive transcranial magnetic stimulation (Matsugi et al., 2017, 2020).

The present study investigated how LVST excitability changes after the repetition of an experimental dynamic balance intervention in which participants stood on a movable plate in the roll plane and kept the plate as parallel to the floor as possible (Sehm et al., 2014). This dynamic balance intervention was chosen as the method of choice to induce the change in LVST excitability, because the intervention required the subject’s head to be kept as stable as possible near the axis of rotation of the plate in response to the acceleration changes applied to the body as the plate moved in the roll plane. We consider that repeated interventions in which the head is constantly subjected to lateral acceleration stimuli may be adapted to reduce changes in the acceleration stimuli. We hypothesized that this dynamic balance intervention would increase LVST excitability and reduce head jerk over the course of repeating the task, and that increased LVST excitability would be associated with decreased head jerk.

Vestibular information has been reported to have the most influence on postural control when the subject is standing on an unstable surface with closed eyes (Peterka, 2018). It has been reported that approximately 20% of patients with peripheral vestibular hypofunction fall when standing on a foam rubber with their eyes closed, and that instability in patients with loss of vestibular function is not observed when the eyes are open or on hard floor surfaces, but only when the eyes are closed and on unstable floor surfaces (Horak et al., 1990; Fujimoto et al., 2009). It has also been shown that vestibular rehabilitation improves the vestibulospinal system, thereby increasing the ability to perform standing balance with altered visual and somatosensory perception (Fetter et al., 1990; Horak et al., 1992). We hypothesized that this dynamic balance intervention, which involved controlling the body in response to vestibular input, could also improve postural stability in sensory conditions that are highly dependent on vestibular sense, and that the improvement of postural stability would also be associated with increased LVST excitability. Therefore, we also assessed the change in postural stability when participants stood on the rubber with eyes closed, due to the repetition of the dynamic balance intervention, and examined its relationship with the change in LVST excitability.

## 2. Materials and methods

### 2.1. Study settings and participants

**FIGURE 1:**
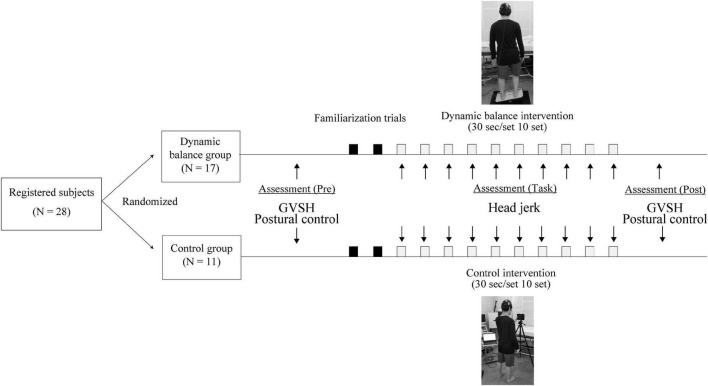
Study design. Both groups performed 10 trials of 30-s interventions, with a 2-min rest in between. GVSH, change rate of the H-reflex amplitude by galvanic vestibular stimulation.

Before conducting the trial, the appropriate sample size was estimated by power analysis using the G*power software (Version 3.1.9.4) (Faul et al., 2007) for two-way analysis of variance (ANOVA). The effect size f was set to 0.6, the alpha error probability to 0.05, the beta error probability to 0.80, the number of groups to two, and the number of measurements to two. The calculated sample size was 24. Therefore, 28 participants were recruited, assuming an expected drop-out of 10%. This study was conducted from January 2020 to March 2020 and included 28 healthy adult volunteers aged 24.8 ± 5.6 years (22 men, 6 women). All participants provided written informed consent to participate in this study in accordance with the Declaration of Helsinki. This study was registered with University Hospital Medical Information Network (identification number: 000037913) and approved by the Ethics Committee of the Nara Medical University Hospital (approval no. 2358). The participants were 1.72 ± 0.62 m tall and weighed 62.1 ± 6.8 kg. All the participants’ dominant foot for kicking a ball was the right. The participants had no history of vertigo disease, no obvious abnormalities in the inner, middle, outer, or intracranial ears no neurological diseases, and were not undergoing treatment for any orthopedic diseases. In addition, none of the subjects had previous experience performing the balance intervention used in this study. All participants were randomly assigned to the dynamic balance group (*n* = 17) or control group (*n* = 11) by a simple randomization method using shuffled cards. The participants performed a dynamic balance intervention or a control intervention, and LVST excitability and static standing stability were assessed before and after the tasks. LVST excitability and static standing stability were measured in random order without rest after the task completion. Head jerk was measured to evaluate changes in head stability over the course of the intervention (Figure 1).

### 2.2. Interventions and head jerk measurements

The dynamic balance intervention required participants to balance on a horizontally unstable board (DYJOC board; SAKAI Medical, Tokyo, Japan) and try to keep it in a horizontal position for as long as possible. The DYJOC board had dimensions of 300 mm × 500 mm × 30 mm and were attached with two semi-circular bosses (φ 80 × 60 mm) behind the platform to limit the tilt movement to a range of 15 degrees to the left and right in the medial-lateral direction. The participants were instructed to stand on the board with their feet 20 cm apart around the axis of board rotation, at a 0 degree toe-out angle for each foot. The participants were asked to fix their gaze on a focal point placed 1 m in front of them, which was adjusted to eye level for each participant. First, participants were given two familiarization trials and allowed to grab a handrail that was mounted in front of them as often as needed to adapt to the movement pattern necessary for the balance task performance (Sehm et al., 2014). Then, participants performed 10 trials, 30 s each, without using a handrail with a 2-min break between trials to avoid fatigue. We used a discovery learning approach (Orrell et al., 2006) in which no information about the performance strategy was provided during the task. The examiner was always ready to assist from behind to prevent falls.

For the control intervention, participants stood in the same foot position as in the dynamic balance intervention on firm ground while performing a maze task with their right index finger on a screen in front of them at the participant’s eye level. The maze task employed in this study is a two-dimensional maze that is navigated through a single passageway from the entrance to the exit. Upon the participant’s arrival at the destination, a subsequent task is presented. This intervention was chosen to allow for fewer head movements, in contrast to the dynamic balance intervention. As in the dynamic balance group, participants performed 10 trials, 30 s each, with a 2-min break between trials.

Participants in the dynamic balance group and control group wore headgear with an accelerometer (UMJG6, UNIMEC, Tokyo, Japan) on their head during the intervention. The line connecting the bilateral auricles was set to be the x axis of the accelerometer. The sampling frequency of the accelerometer was 1,000 Hz. The head jerk was calculated by differentiating the head acceleration data. The root mean square (RMS) of the head jerk was calculated by averaging the data of the x-axis data during the middle 20 s of the task. Previous studies have used head jerk as a measure of effectiveness in complex standing tasks (James, 2014), and it has been observed that x axis head jerk is greater in elderly individuals with lower gait ability and a higher risk of falls compared to younger subjects, suggesting that it is a valid indicator of postural stability (Brodie et al., 2014). In this study, because the task used an unstable plate along the x axis, calculations were performed using only mediolateral axis acceleration data.

### 2.3. Assessment of the LVST excitability

The participants were assessed using the unconditioned soleus H-reflex followed by the conditioned soleus H-reflex by the GVS in the prone position before and after a block of 10 trials of either the dynamic balance or control intervention. The participants adopted the prone position on a padded bed with his or her head facing forward, with the upper limbs at the sides of the body. The subject’s foot was immobilized in an orthotic to minimize ankle movement, while maintaining the ankle position at 90^°^ (measured as the internal angle between the line connecting the fibula head to the lateral metatarsal and the fifth metatarsal) and the knee angle at approximately 15^°^ (measured as the angle between the femur and the line connecting the fibula to the lateral metatarsal). The subjects were instructed to keep their head and upper limbs in a stable position and eyes closed while remaining awake during the experiment to avoid confounding the results of the H-reflex before and after the task (Traccis et al., 1987; Kennedy and Inglis, 2002; Okada et al., 2018). Electromyographic activity of the right soleus muscles was recorded using bipolar Ag/AgCl surface electrodes (Vitrode F-150S; Nihon Kohden, Tokyo, Japan) placed 2 cm apart on the right soleus muscle. A ground electrode was positioned on the medial malleolus of the same leg. The electromyographic signals were amplified by a computerized modular electromyographic detection unit (Neuropack X1, Model MEB-2306; Nihon Kohden, Tokyo, Japan) with a 15–3 kHz bandpass filter, converted to digital signals, and stored on a personal computer.

The H-reflex was recorded from the right soleus muscle. Ten consecutive soleus H-reflexes were evoked by electrically stimulating the tibial nerve at the popliteal fossa at varying time intervals between 3 and 5 s. The stimulation electrode was a ball fixed to the leg with a strap so that constant stimulation could be applied to the tibial nerve. The duration of the stimulation was 1 ms. We assessed the right soleus H-reflex amplitude at a stimulus level that produced an M-wave equal to 5–25% of the maximum M-wave to adjust for the ascending limb of the H-reflex recruitment curve (Kennedy and Inglis, 2001; Okada et al., 2018).

The conditioned H-reflex was assessed by providing the GVS 100 ms preceding the tibial nerve stimulation to evoke the soleus H-reflex (Kennedy and Inglis, 2001). GVS was delivered using the SEN-3401 stimulator connected to an isolator (SS-104; Nihon Kohden, Tokyo, Japan) by two Ag/AgCl surface electrodes (Vitrode F-150s, 18 × 36 mm; Nihon Kohden, Tokyo, Japan), with the cathode located over the right mastoid and the anode located over the left mastoid process. The GVS consisted of a 200-ms square-wave pulse, and the intensity was set at 3-mA (Okada et al., 2018).

The peak-to-peak amplitudes of the H-reflex were computed offline. The GVSH was calculated using the following formula: *(conditoned H reflex ampulitued [mV] ÷ unconditoned H reflex ampulitued [*mV*]−1)×100%)* (Lowrey and Bent, 2009; Nakamura et al., 2021). The change rate of the H-reflex by the GVS reflected LVST excitability (Horak et al., 1992).

### 2.4. Posturography

All participants used a force plate (Gravicorder G5500; Anima, Tokyo, Japan) to measure posture before and after a block of 10 trials of either the dynamic balance or control intervention on a foam rubber with their eyes closed at a sampling frequency of 20 Hz. The foam rubber (Anima, Tokyo, Japan) was made of natural rubber, with a tensile strength of 2.1 Kgf/cm^2^, elongation stretch percentage of 100%, density 0.162 g/cm^2^, and thickness of 3.5 cm. The participants remained in the standing position for 60 s with the distal ends of the toes 30° apart, and the heels of both feet were close to each other. The participants were instructed to face forward without turning their heads and to keep their arms at their sides. The mean velocity of center of pressure (COP) movement (COP velocity) and the outer circumference area of the envelope traced by COP movement (COP area) were computed. The scaling exponent α for each of the x and y axis was also computed to investigate the long-range correlations within the time series of the CoP trajectories. The scaling exponent α was calculated by using a fractal analysis method for biological time signals called detrended fluctuation analysis (DFA) (Peng et al., 1995). In a first step, mean is subtracted from the original time series, which is the integrated:


$$y\,\left(k\right)=\sum_{i=1}^{k}\left[x\,\left(i\right)-\bar{x}\right]$$

This integrated series is then divided into windows of equal length *n* ranging from 4 to *N*/4 data points. The local trend of each window *y*_*n*_ is obtained and subtracted from the summed series by using a least-squared fit to obtain the detrended fluctuation *F*(*n*):


$$F\,\left(n\right)=\sqrt{\frac{1}{N}\,\sum_{k=1}^{N}{\left[y\,\left(k\right)-y\,\_\,n\right]}^{2}}$$

The scaling exponent α is the slope of a double logarithm plot of *F(n)* vs. *n* (Schniepp et al., 2013).

Previous studies have reported intraclass correlation coefficients of 0.87 for pitch and 0.66 for roll in postures with closed eyes and no head movement on the foam rubber (Alsubaie et al., 2019). Therefore, posturography was measured once before and after each intervention in this study in order to minimize the decay of the effect of the dynamic balance intervention as much as possible.

### 2.5. Statistical analyses

The Shapiro–Wilk test was used to assess the normality of all variables. Data are presented as the mean values and standard deviation of the mean. Participant characteristics were compared between the groups using non-paired *t*-tests (numerical data: age, height, and weight) and Fisher’s exact test (nominal data: sex). When normality was found, a two-way repeated measures analysis of variance (ANOVA) was performed to test the change in head jerk during the task by factors (group × trial) and to test the difference in the change in GVSH, COP area, and COP velocity by factors (group × time). If normality was not found, a Friedman test was performed for each group; if head jerk showed significant differences between trials, Dunn’s test was used to evaluate group differences between the first and other trials. A *post hoc* analysis of the change in GVSH, COP area, and COP velocity was performed using Bonferroni correction. Spearman’s rank correlation coefficient was used to examine the relationship between the change in GVSH and the change in COP area and COP velocity, or a change in head jerk from trials 1 to 10 for each group. Only in the dynamic balance group, first-time head jerk and GVSH changes were examined using Spearman’s rank correlation coefficient. Statistical analyses were performed using GraphPad Prism ver.8.00 for Windows, GraphPad Software, La Jolla California USA. The significance level was set at *p* < 0.05.

## 3. Results

All participants completed all assessments and interventions, and no adverse effects were observed. Only head jerk was not found to be normally distributed; all other variables followed a normal distribution. There were no significant differences in the participant characteristics between the two groups (age: dynamic balance group 24.0 ± 5.5 years, control group 26.0 ± 5.4 years, *p* = 0.37; height: dynamic balance group 1.70 ± 6.2 cm, control group 1.74 ± 5.6 cm, *p* = 0.17; weight: dynamic balance group 61.9 ± 7.9 kg, control group 62.5 ± 4.7 kg, *p* = 0.83).

**FIGURE 2:**
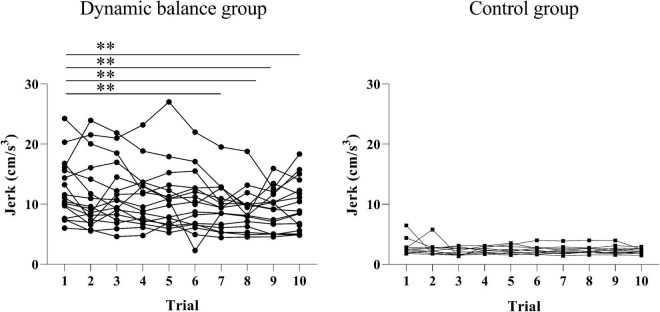
Changes in head jerk during the intervention for each group. The head jerks for each trial across all subjects were graphed. There is a significant decrease in head jerk in the dynamic balance group in trials 7, 8, 9, and 10 compared to trial 1. Black points denote the dynamic balance group; black squares denote the control group; A double asterisk indicates a *p*-value of less than 0.01.

There was a significant change in head jerk between the 10 trials only in the dynamic balance group (*p* < 0.001; trial 1: 12.45 ± 4.95 cm^3^, trial 2: 11.45 ± 5.73 cm^3^, trial 3: 11.69 ± 5.18 cm^3^, trial 4: 10.92 ± 4.80 cm^3^, trial 5: 10.94 ± 5.39 cm^3^, trial 6: 10.23 ± 4.89 cm^3^, trial 7: 9.48 ± 3.71 cm^3^, trial 8: 9.03 ± 3.45 cm^3^, trial 9: 9.15 ± 3.36 cm^3^, trial 10: 9.89 ± 4.17 cm^3^**)** and no change in the control group (*p* = 0.783; trial 1: 2.86 ± 1.40 cm^3^, trial 2: 2.61 ± 1.14 cm^3^, trial 3: 2.13 ± 0.64 cm^3^, trial 4: 2.34 ± 0.50 cm^3^, trial 5: 2.34 ± 0.66 cm^3^, trial 6: 2.34 ± 0.68 cm^3^, trial 7: 2.30 ± 0.68 cm^3^, trial 8: 2.34 ± 0.66 cm^3^, trial 9: 2.37 ± 0.70 cm^3^, trial 10: 2.22 ± 0.43 cm^3^). The *post hoc* analysis indicated that head jerk significantly decreased only in the dynamic balance group in trials 7, 8, 9, and 10 compared to trial 1 (trial 1 vs. trial 7: *p* = 0.004; trial 8: *p* < 0.001; trial 9: *p* < 0.001; trial 10: *p* = 0.013), as illustrated in Figure 2.

**FIGURE 3:**
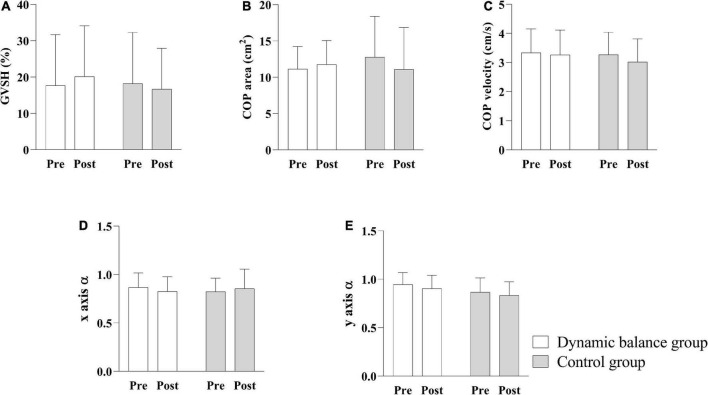
Comparison of GVSH, COP area, COP velocity, x axis scaling exponent α, and y axis scaling exponent α pre- and post-intervention. The interaction between group and time is not significant, and there is no significant main effect of group on GVSH **(A)**, COP area **(B)**, COP velocity **(C)**, x-axis scaling exponent α **(D)**, and y-axis scaling exponent α **(E)**. White bars indicate the mean of the dynamic balance group; gray bars indicate the mean of the control group; and error bars indicate standard errors. GVSH, change rate of the H-reflex amplitude by galvanic vestibular stimulation; COP, center of pressure.

No significant interaction was observed between group and time for GVSH [*F*(1, 26) = 0.666, *p* = 0.442], COP area [*F*(1, 26) = 3.50, *p* = 0.073], COP velocity [*F*(1, 26) = 0.888, *p* = 0.355], x axis α [*F*(1, 26) = 0.930, *p* = 0.3439], or y axis α [*F* (1, 26) = 0.030, *p* = 0.865] nor was there a significant main effect of group [GVSH: *F*(1, 26) = 0.102, *p* = 0.752; COP area: *F*(1, 26) = 0.095, *p* = 0.760; COP velocity: *F*(1, 26) = 0.258, *p* = 0.620; x axis α : *F*(1, 26) = 0.023, *p* = 0.881; y axis α :*F* (1,26) = 2.982, *p* = 0.096] or time [GVSH: *F*(1, 26) = 0.036, *p* = 0.851; COP area: *F*(1, 26) = 0.736, *p* = 0.399; COP velocity: *F*(1, 26) = 2.786, *p* = 0.107; x axis α : *F*(1, 26) = 0.021, *p* = 0.887; y axis α : *F*(1, 26) = 1.555, *p* = 0.224], as illustrated in Figure 3. However, 47.1% (8/17) of subjects in the dynamic balance group and 0% (0/11) in the control group displayed a change in GVSH greater than 11%, the MDC established in a previous study (Nakamura et al., 2021).

**FIGURE 4:**
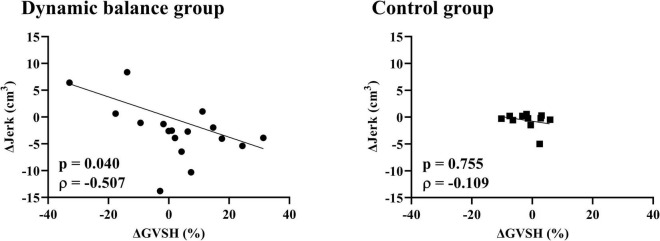
Relationship between changes in GVSH and head jerk in each group. There is a negative correlation between the pre- and post-intervention changes in GVSH and the change in trial 1 and 10 of head jerk in the dynamic balance group only. Black points denote individual data points for the dynamic balance group, and black squares denote individual data points for the control group. Straight lines indicate robust regression. GVSH, change rate of the H-reflex amplitude by galvanic vestibular stimulation; Δjerk, difference between head jerk on trial 1 and on trial 10.

**FIGURE 5:**
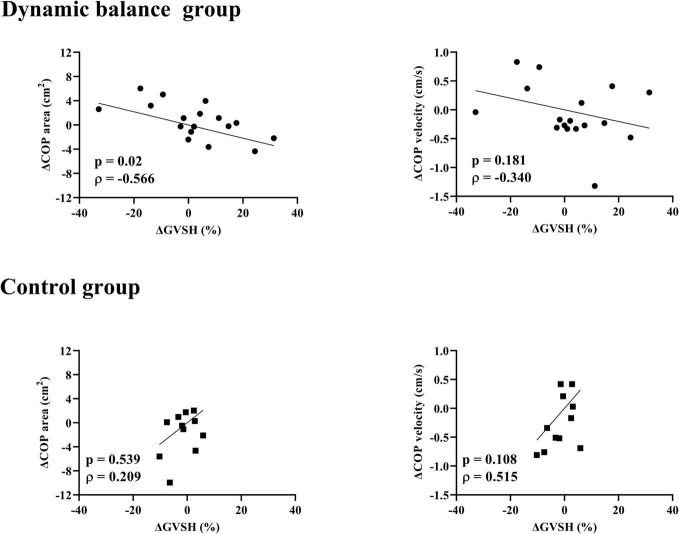
Relationship between changes in GVSH and COP area, COP velocity in each group. There is a negative correlation between the pre- and post-intervention changes in the GVSH and COP-area-only for the dynamic balance group. Black circles indicate individual data points for the dynamic balance group; black squares indicate individual data points for the control group. Straight lines indicate linear regression. GVSH, change rate of the H-reflex amplitude by galvanic vestibular stimulation; COP, center of pressure.

There was a negative correlation only in the dynamic balance group between the change in GVSH and the change in head jerk from trials 1 to 10 (Figure 4), and between the changes in GVSH and COP area before and after the task (Figure 5). No significant correlation was observed between the changes in GVSH and COP velocity before and after the tasks in either group. In the dynamic balance group, there was a significant positive correlation between the initial head jerk and the change in GVSH (Table 1). All data are available online from data storage.^1^

**TABLE 1:** Relationship between initial head jerk and changes in GVSH, COP area COP velocity in dynamic balance group.

	Change in GVSH	Change in COP area	Change in COP velocity
Initial head jerk	***p* = 0.044**	*p* = 0.104	*p* = 0.235
	**ρ = 0.488**	ρ = −0.409	ρ = −0.303

This table presents the relationships between the head jerk on the first trial of intervention and the change in GVSH and COP before and after the intervention for the subjects in the dynamic balance group. There was a significant positive correlation between the initial head jerk and the change in GVSH. GVSH, change rate of the H-reflex amplitude by galvanic vestibular stimulation; COP, center of pressure.

Items for which significant correlations were found are highlighted in bold.

## 4. Discussion

The present study is the first to examine the changes in LVST excitability in a prone position and postural sway when standing on a foam rubber with eyes closed before and after a dynamic balance intervention in young healthy participants. The main findings of this study were as follows: (1) head jerk significantly reduced over the trials of the dynamic balance intervention; (2) GVSH, COP area, and COP velocity did not significantly change after the dynamic balance intervention or control intervention, and (3) the changes in GVSH showed significant negative correlations with changes in COP area after the intervention and changes in head jerk during the intervention only in the dynamic balance group.

### 4.1. Head jerk decrease over the course of the dynamic balance intervention

Head jerks decrease over the course of the dynamic balance intervention, but no change was observed in the control group. It is possible that the dynamic balance intervention used in this study caused an immediate adaptation to stabilize the head. Although the dynamic balance task required the participants to keep the unstable board horizontal, we believe that the strategy to stabilize the head in sensorimotor adaptation was used because of the amount of stimulation to the head. However, the head jerk was 12.45 ± 4.95 cm^3^ in Trial 1 and 9.89 ± 4.17 cm^3^ in Trial 10, so the subject’s adaptation was not a constant response with large individual differences. Drawing individual plots, subjects with higher head jerk at the start tended to show adaptation to head stabilization, suggesting that vestibular stimulation may have provided sensory feedback. It is well-established that somatosensory weighting in postural control is diminished on unstable floor surfaces in healthy individuals (Peterka, 2018), and it is possible that vestibular sensory feedback contributed to the observed adaptation in this study.

### 4.2. Changes in GVSH and COP with intervention

However, there was no significant change in GVSH, COP area, COP velocity, x axis scaling exponent α, and y axis scaling exponent α pre- and post- intervention between the two groups. The dynamic balance intervention used in this study was thought to immediately increase head stability, but the change may have been a task-specific learning effect. Head jerk in the dynamic balance group had a large standard error, and there may have been a large variation in adaptation to the task among the participants. The control group in this study also performed a dual task of standing and cognitive tasks. Previous reports have shown that the dual task improves postural control (Nafati and Vuillerme, 2011; Kümmel et al., 2016), which may have influenced the performance of the control group, but since there was no main effect for any of the items, we believe that there was little improvement in both groups. The participants in this study were healthy young adults, and they did not have problems with visual, somatosensory, or vestibular perception, which are necessary for postural control, and any sensory weighted adaptation was possible, leading to variability in the results. The significant positive correlation between the initial head jerk and changes in GVSH also suggests that some participants had low adaptation effectiveness due to head stability during the dynamic balance intervention from the start, which may have led to no change in GVSH or COP. It is possible that the dynamic balance intervention could have increased the excitability of the LVST if only those participants with a high level of head jerks at the start of the dynamic balance intervention had been included. In addition, it has been reported that there is no relationship between static and dynamic postural stability, and that balance training for healthy subjects produces task-specific changes that do not transfer to other tasks (Sell, 2012; Kümmel et al., 2016). It is possible that head stability was increased during the dynamic balance intervention in the present study, but there was no transfer to static postural control ability. In this study, the intervention period was short (only 1 day), and GVSH and COP measurements were performed randomly in the post-intervention evaluation, which may have attenuated the effect during the time elapsed between the intervention and the measurement, preventing an adequate evaluation of the intervention effect.

### 4.3. Correlation between changes in GVSH and COP

The correlation between the pre- and post- intervention changes in the GVSH and COP areas showed a significant negative correlation in the dynamic balance group, suggesting that an increase in GVSH results in a smaller area of sways in the standing position on the foam rubber with eye closed. To date, no experimental studies have been identified that investigate the relationship between LVST excitability and postural control in humans, making the present finding of particular significance in demonstrating the validity of GVSH as an assessment method. Although the method of adaptation in the present study differed among participants, those with a greater tendency to increase LVST excitability tended to have less postural sway while standing on the rubber foam, where vestibular information has been reported to influence postural control (Fujimoto et al., 2009; Peterka, 2018). The posture reactions of normal participants to visual, vestibular, and proprioceptive stimulation are quite variable (Bonan et al., 2013), suggesting that although adaptation to the dynamic balance intervention in the present study resulted in immediate changes in GVSH, the adaptive strategies varied greatly among the participants and excitability was not uniformly enhanced. This correlation was not found in the control group, and the change in GVSH in the control group was smaller than that in the dynamic balance group. In our previous study, the minimal detectable change of GVSH was shown to be 11.0 % (Nakamura et al., 2021). In the current study, 47.1% of participants in the dynamic balance group displayed a change in GVSH greater than the MDC. In contrast, none of the participants in the control group exhibited such a change. These changes occurred within the margin of measurement error, and it is possible that the lack of change in LVST excitability due to the intervention resulted in the absence of a correlation. Alternatively, it may be that the vestibular sensory contribution to postural control was minimal in the control group, as there was no change in head jerk between trials. We observed that there was no significant correlation between the changes in GVSH and COP velocity in either group. It has been reported that the COP area and COP velocity are larger for patients with vestibular disorders than in healthy participants, which contradicts the results of this study. For dynamic balance intervention in this study, the participants were instructed to keep the board horizontal as much as possible, and it is an intervention that encouraged voluntary postural control. It is known that the mean COP velocity increases when postural control is exerted effortfully in healthy participants (Ueta et al., 2015), and it is possible that immediate adaptation did not correlate with other factors of voluntary postural control besides LVST excitability. Some intervention studies of patients with peripheral vestibular disorders have shown improvement in standing maximum amplitude angle as an outcome (Fetter et al., 1990; Horak et al., 1992; Morisod et al., 2018), suggesting that the function of the LVST may contribute to spatial localization.

### 4.4. Correlation between changes in GVSH and head jerk

We investigated the relationship between changes in head jerk in trials 1 and 10 and changes in GVSH to examine whether changes in head stability during the intervention produced immediate changes in LVST excitability. We found a moderate negative correlation, indicating that a decrease in head jerk during the intervention was associated with an increase in the GVSH only in the dynamic balance group. The participants who adopted the strategy of increasing head stability while repeating the intervention showed an immediate increase in LVST excitability. There was a significant positive correlation between initial head jerk and change in GVSH. Participants were performing the dynamic balance intervention in this study for the first time, but there was a difference in performance at the start. Participants with less stable heads and greater changes in head acceleration stimuli were more likely to experience adaptation phenomena, indicating that adaptation may affect changes in LVST excitability. It is likely that the adaptation to the dynamic balance intervention observed in the present study was mediated by vestibular sensation as a feedback stimulus, with the LVST likely playing a significant role. We posit that the modification of postural control strategies through vestibular feedback stimulation resulted in reduced head jerk, and that changes in GVSH, which reflect the excitability of the LVST involved in adaptation, were correlated with changes in head jerk. In addition, in a study that measured structural changes in the brain by magnetic resonance imaging during a dynamic balance intervention session (similar to this study) performed for 90 min once a day for 2 weeks, an increase in gray matter in the premotor and inferior parietal areas was observed. This region is known to be associated with complex motor skill acquisition and the integration of vestibular signals for postural control (Taubert et al., 2010). Although the duration of the intervention in this study was short compared to previous studies, the adaptive phenomenon of the central vestibular system to the dynamic balance intervention may have influenced the change in GVSH.

### 4.5. Study limitations and future research directions

The present study has several limitations. First, the adaptive effect on the intervention was evaluated only using head jerk. Since the intervention was to keep an unstable board horizontal, the measurement of the board angle may have been directly related to the learning effect. However, we focused on the effect of vestibular function, thus, we considered measuring head jerk would have demonstrated a learning effect. In addition, head jerk was appropriate for comparing the amount of head stimulation during the intervention with the control condition. Second, GVSH was measured only with the right foot in the prone position. Although it has been reported that there is no difference in GVSH between right and left in healthy participants (Nakamura et al., 2021), it is known that there is a relationship between the left and right ratio and the static standing position, and adaptation to the intervention may differ between the left and right. In addition, to examine the relationship with posture control, it is possible that the measurement in the sitting position (Tanaka et al., 2021), an antigravity limb position, is more sensitive for observing the changes. Third, this study used a small sample size and a simple randomization technique, which resulted in a difference in the number of subjects included in each group. This may have affected the results, and it is therefore necessary to increase the number of samples and make the number of participants equal between the groups in the future. Fourth, the force plate used in this study could only sample at a maximum of 20 Hz, which is lower than the sampling frequency used in many postural control studies. The sampling frequency may have affected the results. Finally, this study only measured immediate changes and could not explain the long-term improvement in LVST excitability by vestibular rehabilitation. The long-term effects of vestibular rehabilitation will be investigated in future studies.

## 5. Conclusion

LVST excitability did not change consistently by the dynamic balance intervention for the healthy young adult participants, although increased LVST excitability may have been associated with improved head stability during the dynamic balance intervention and improved postural stability in sensory conditions with high vestibular sensory weighting after the intervention. Future studies are required to examine the long-term effects of continued dynamic balance exercise or vestibular rehabilitation on LVST excitability.

## Data availability statement

The original contributions presented in this study are included in the article/supplementary material, further inquiries can be directed to the corresponding author.

## Ethics statement

The studies involving human participants were reviewed and approved by the Ethics Committee of the Nara Medical University Hospital (approval no. 2358). The patients/participants provided their written informed consent to participate in this study.

## Author contributions

TS and YO designed the study. TS and MM collected the data. TS analyzed the data and initially wrote the manuscript. TS, YO, JN, KU, HT, and TK interpreted the data. All authors revised the manuscript and approved the submitted version.

## Footnotes

1
http://dx.doi.org/10.17632/kbncyd8fk5.1
